# Main regulatory pathways, key genes and microRNAs involved in flower formation and development of moso bamboo (*Phyllostachys edulis*)

**DOI:** 10.1111/pbi.12593

**Published:** 2016-07-16

**Authors:** Wei Ge, Ying Zhang, Zhanchao Cheng, Dan Hou, Xueping Li, Jian Gao

**Affiliations:** ^1^ Key Laboratory of Bamboo and Rattan Science and Technology of the State Forestry Administration International Centre for Bamboo and Rattan Beijing China; ^2^ China National Engineering Research Center for Information Technology in Agriculture Beijing China

**Keywords:** moso bamboo, flower formation and development, regulatory pathway, Dof, MADS‐box, microRNA

## Abstract

Moso bamboo is characterized by infrequent sexual reproduction and erratic flowering habit; however, the molecular biology of flower formation and development is not well studied in this species. We studied the molecular regulation mechanisms of moso bamboo development and flowering by selecting three key regulatory pathways: plant–pathogen interaction, plant hormone signal transduction and protein processing in endoplasmic reticulum at different stages of flowering in moso bamboo. We selected *PheDof1*,* PheMADS14* and six microRNAs involved in the three pathways through KEGG pathway and cluster analysis. Subcellular localization, transcriptional activation, Western blotting, *in situ* hybridization and qRT‐PCR were used to further investigate the expression patterns and regulatory roles of pivotal genes at different flower development stages. Differential expression patterns showed that *PheDof1*,* PheMADS14* and six miRNAs may play vital regulatory roles in flower development and floral transition in moso bamboo. Our research paves way for further studies on metabolic regulatory networks and provides insight into the molecular regulation mechanisms of moso bamboo flowering and senescence.

## Introduction

Moso bamboo (*Phyllostachys edulis*) is a kind of large woody bamboo with great ecological, cultural and economic values of all bamboos in Asia covering about 3 000 000 hm^2^, accounting for 20% of the total forest area in the world (Li *et al*., 2012). In China, it covers 6.01 million hm^2^, and the total annual forest production was valued at 5 billion US dollars in 2013. Moso bamboo is a perennial plant characterized by rapid growth and a long vegetative stage that lasts for decades or even longer before flowering (Lin *et al*., 2010). However, it always flowers synchronously followed by widespread death in a large area, limiting the development of the moso bamboo industry. Peng *et al*. (2013) had pointed out that 30% of flowering‐related genes including DOF and MADS‐box families were heat shock protein genes, stress‐related genes and transcription factors. An increase expression of Dof under drought stress induced MADS‐box expression, which in turn promoted flowering in moso bamboo. As Dof also affected the expression of the floral integrator *Heading date3a* (*Hd3a*) gene, Gao *et al*. (2014) suggested that an active Dof‐Hd3a‐MADS‐flowering pathway might significantly affect flowering in Moso bamboo. Moreover, we have successfully collected full‐scale moso bamboo flowering samples through several year endeavour of our research group and used the paraffin section technique to study moso bamboo inflorescence morphogenesis for the first time. A large number of differentially expressed microRNAs and their targets participated in diverse primary biological pathways and play significant regulatory roles in moso bamboo flowering (Gao *et al*., 2015). However, it is different from the classic flowering regulation pathway based on our research data, such as no significant change in expression of *CO* during moso bamboo flowering. To propagate moso bamboo plants, it is imperative to determine the signalling pathways involved in flowering and the respective genes.

Dof (DNA binding with one zinc finger) transcription factors are a group of plant‐specific transcription factors. The cDNA sequence of the first Dof was obtained from *Zea mays* (Yanagisawa and Izui, 1993). Since then, many Dof genes have been cloned from various plant species including 37 Dof genes from *Arabidopsis thaliana* (Ward *et al*., 2005), 30 from *Oryza sativa* (Li *et al*., 2009; Lijavetzky *et al*., 2003) and 28 Dof genes from *Glycine max* (Wang *et al*., 2007). Dof transcription factors are involved in many biological processes in plant growth and development. *JcDof3* is a biological clock gene that regulates the flowering time in *Jatropha carcass* (Yang *et al*., 2011). The overexpression of *OsDof12* in *O. sativa* induces early flowering under long‐day or drought stress conditions (Li *et al*., 2009).

The MADS‐box family members that were identified originally as floral homeotic genes are significant transcription factors for plant development (De Folter and Angenent, 2006; Kaufmann *et al*., 2005). These genes were initially characterized from *Saccharomyces cerevisiae* (Passmore *et al*., 1988) and *Arabidopsis* (Yanofsky *et al*., 1990). MADS‐box genes participate in floral organ development and flowering time regulation (Theissen *et al*., 2000). Thirty‐four MADS‐box genes were identified in *O. sativa*, 15 of which regulate flower development (Lee *et al*., 2003). *OsMADS18* regulates the differentiation of shoot apical meristem and causes early flowering in *O. sativa* (Fornara *et al*., 2004) while *OsMADS3* and *OsMADS58* control carpel formation (Dreni *et al*., 2007). Mutants of MADS‐box genes such as *cfo1* (Sang *et al*., 2012) can change the morphology of floral organs.

miRNAs are small regulatory RNAs that are involved in a number of processes including growth and development control, cell differentiation, phytohormone signals, abiotic and biotic stress (Liu and Chen, 2009; Zhu *et al*., 2008). Many miRNAs target transcription factors have been implicated in key regulatory pathways (Jones‐Rhoades *et al*., 2006). Yu *et al*. (2012) showed that Gibberellins regulate flowering via miR156 targeted squamosa promoter binding‐like (SPL) transcription factors. Millar and Gubler (2005) suggested that miR159 was related to the development and morphology of clinandriums. miR164 targeted NAM, ATAF1/2 and CUC2 domain containing transcription factors (NAC) and specify extraordinary cell types at the later stages of flower development (Nag and Jack, 2010). The expression levels of miR164a showed significant down‐regulated trend from no flowering leaves to flowering samples in moso bamboo, negatively correlated with that of its target (Gao *et al*., 2015). Other flowering‐related miRNAs include miR166, which affects the morphogenesis of flowers (Jung and Park, 2007) and miR167, which regulates the development and maturity of stamens and pistils (Nagpal *et al*., 2005). In this study, we aimed to select key regulatory pathways at different stages of floral development in moso bamboo. We used bioinformatics analysis to identify three regulatory pathways, transcription factors and the miRNAs involved in the regulatory pathways and studied the expression patterns of key genes using Western blotting, *in situ* hybridization and qRT‐PCR. Our research reveals the molecular regulation mechanism of moso bamboo flowering and provides important information for selecting significant genes to study their function in floral development.

## Results

### Pathway function annotation of differentially expressed genes (DEGs)

**Figure 1:**
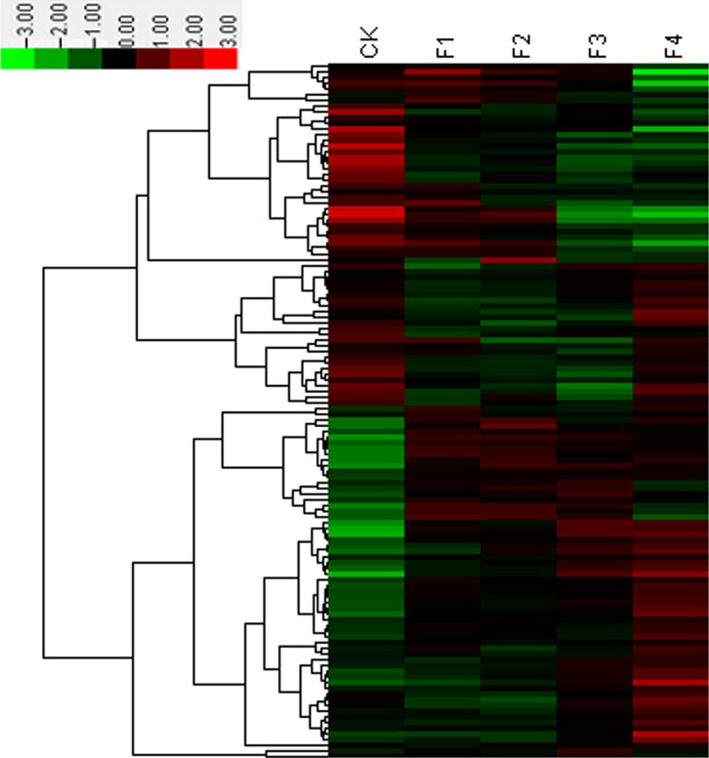
Hierarchical cluster analysis of DEG gene expression based on log ratio RPKM data.

Our results following differential gene expression (DGE) analysis showed that 11 703 differentially expressed genes changed significantly during the four stages of flower formation and development. These genes were annotated with KEGG pathway and were involved in 128 metabolic pathways. The RPKM of each metabolic pathway was calculated, and cluster analysis was carried out with the logarithm of RPKM (Figure 1). We used cluster analysis to identify the two major gene groups that were up‐ and down‐regulated. The highest expressional pathways in the up‐regulated groups were plant–pathogen interaction, plant hormone signal transduction and protein processing in endoplasmic reticulum.

**Figure 2:**
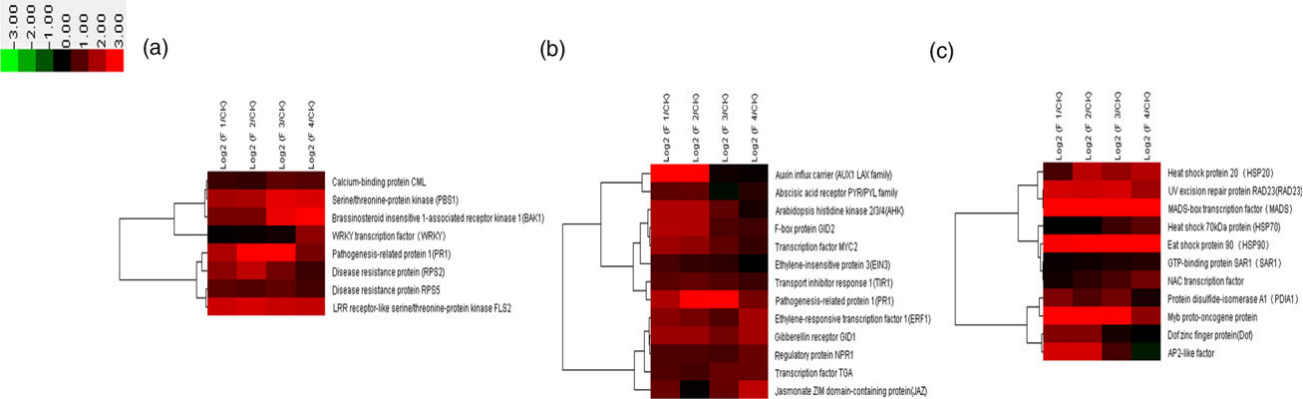
Differential expression analysis of related genes involved in main regulatory pathways. Heatmap for clustering analysis of related genes. The bar represents the scale of the expression levels of the genes (log 2). CK: nonflowering moso bamboo leaves, F1: floral bud formation stage, F2: inflorescence growing stage, F3: bloom stage, F4: embryo formation stage. (a) Differential expression analysis of related genes involved in plant–pathogen interaction. (b) Differential expression analysis of related genes involved in plant hormone signal transduction. (c) Differential expression analysis of related genes involved in protein processing in endoplasmic reticulum.

**Figure 3:**
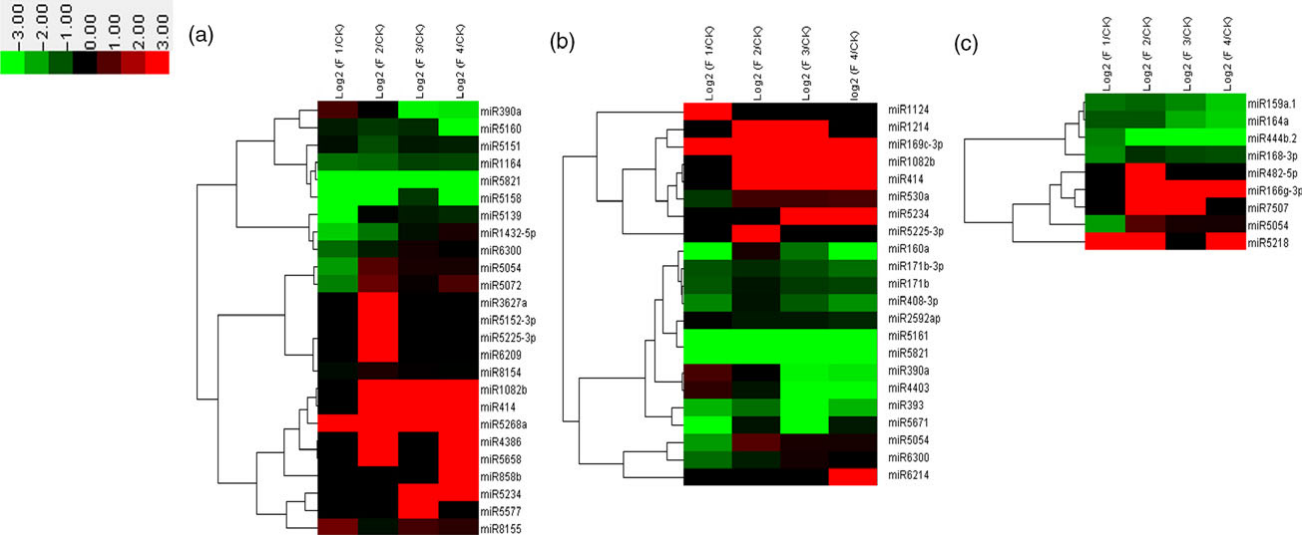
Differential expression analysis of related miRNAs involved in main regulatory pathways. Heatmap for clustering analysis of related miRNAs. The bar represents the scale of the expression levels of the genes (log 2). (a) Differential expression analysis of related miRNAs involved in plant–pathogen interaction. (b) Differential expression analysis of related miRNAs involved in plant hormone signal transduction. (c) Differential expression analysis of related miRNAs involved in protein processing in endoplasmic reticulum.

Our results showed that 269 differentially expressed genes were involved in plant–pathogen interaction (Figure 2). Plant disease resistance genes FLS2, PR1, RPS2 and RPS5 showed high expression levels during the whole flower formation and developmental process. CML, PBS1 and BAK1 showed higher expression at later stages of flower formation and development compared with that at early stages (Figure 2a). The expression of WRKY gene which is related to plant senescence increased gradually with the development of the flower. The expression of WRKY was the highest when moso bamboo entered the death stage on completion of embryo development (Figure 2a). The high expression levels indicated that the process of flower formation and development might be a phenotype of moso bamboo senescence. We analysed the targets of differentially expressed miRNAs using the KEGG pathway, and miRNAs that putatively play significant regulatory roles in plant–pathogen interaction based on their targets were used to perform cluster analysis (Figure 3a). It showed that miR390a, miR5139 and miR5821 expression levels were lower in flowering samples than those in nonflowering samples (Figure 3a).

Flower formation and development in plants is a cue to enter the reproductive development, and the perception and response of plant hormones plays vital roles in the regulation of flower formation and development. Our results showed that 301 differentially expressed genes were involved in plant hormone signal transduction. Cluster analysis indicated that the expression of IAA was higher in the early stage of flower formation and development and presented down‐regulated trend with flower development (Figure 2b). GA metabolism genes related to flower development such as *GID1* and *GID2* showed high expression levels at four different periods of flower development. Thus, the genes related to GA metabolism played a crucial role in whole flower formation and development. Moreover, the expression of genes involved in jasmonic acid and salicylic acid metabolism and related to stress, including JAZ, transcription factor MYC2 and TGA, was significantly up‐regulated at floral bud formation stage, bloom stage and embryo formation stage. These results showed that the stress pathways and hormone regulatory pathways related to stress resistance were mutually connected, highly expressed and jointly regulated flower development in moso bamboo. However, a number of miRNAs involved in plant hormone signal transduction, for example miR160a, miR171b and miR393, were down‐regulated in the four periods (Figure 3b).

Our results showed that 170 differentially expressed genes were involved in protein processing in the endoplasmic reticulum (ER). Cluster analysis indicated that the expressions of MADS‐box and MYB were markedly up‐regulated during the four flower developmental stages (Figure 2c). Many transcription factors associated with stress resistance were found in protein processing in the ER such as HSP90, which showed significant up‐regulation. HSP90 is associated with hormone receptors, other transcription factors (such as HSP20), stress signalling pathways, intracellular transport and kinase activity regulation. The high expression of HSP90 indicated that the process of flower development in moso bamboo might be related to stress. Dof, the other important stress‐related gene was highly expressed at the floral bud formation stage and inflorescence growing stage, while gradually decreased with the flowers withered and reached the minimum at embryo formation stage (Figure 2c). Therefore, we inferred that Dof mainly played roles at early stages of flower formation and development in moso bamboo; it also participated in the regulation of floral bud formation and elongation. Moreover, the expression levels of AP2, NAC and RAD23 were also significantly up‐regulated in the flower development process (Figure 2c). The putative ER protein processing miRNAs such as miR159a.1, miR164a and miR168‐3p showed lower expression levels in flowering samples were than those in nonflowering leaves (Figure 3c).

### Phylogenetic analysis of PheDof1 and PheMADS14

We found that 15 Dof transcription factors and 34 MADS transcription factors that were involved in protein processing in the ER in moso bamboo inflorescence transcripts. *PheDof1* (PH01000664G0640) and *PheMADS14* (PH01000222G1190) showed significantly high expression levels in the flowering period. Thus, *PheDof1* and *PheMADS14* were selected for further research.

**Figure 4:**
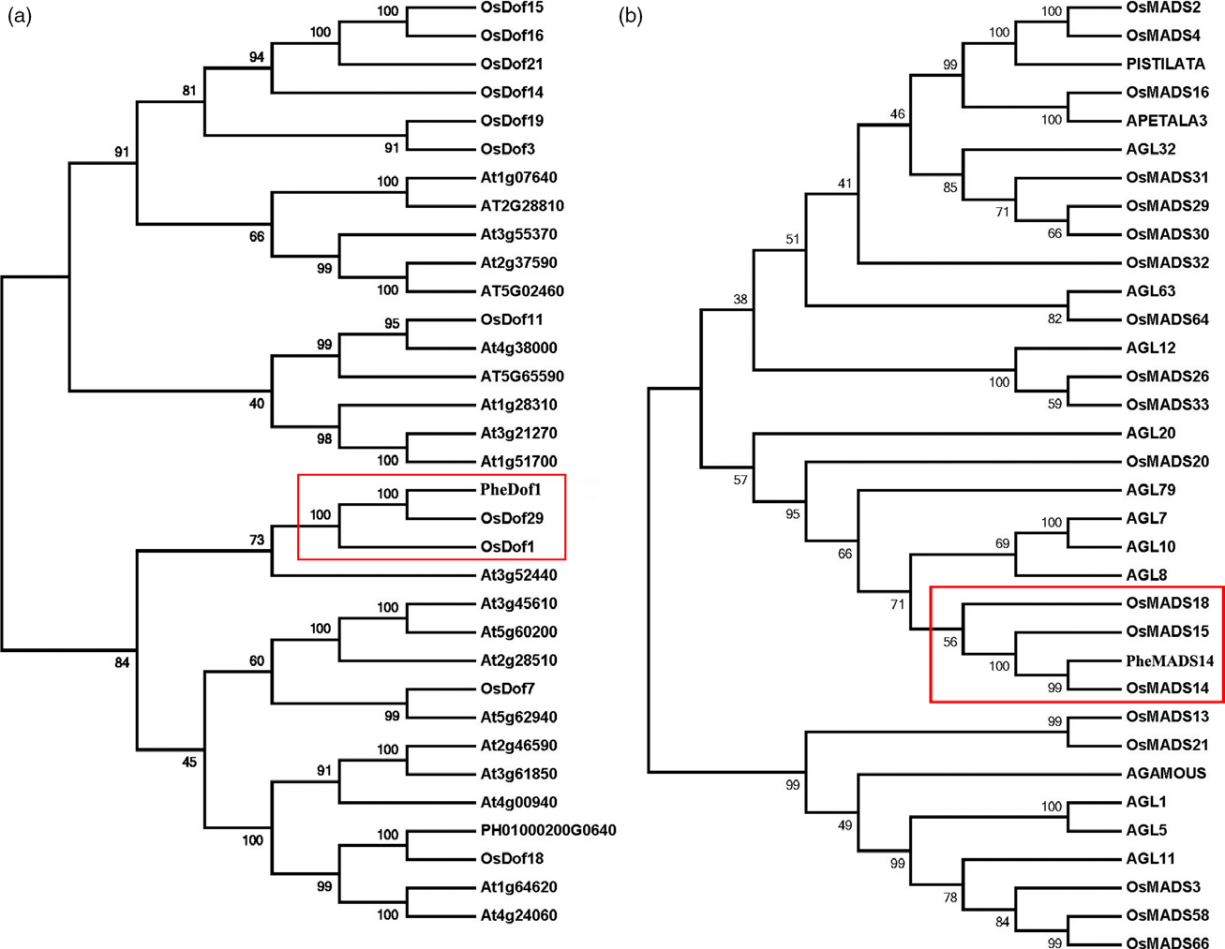
Phylogenetic analysis of *PheDof1* and *PheMADS14*. (a) Joined phylogenetic tree of moso bamboo *PheDof1* (PH01000200G0640) and Dof transcription factors of *Arabidopsis* and *O. sativa*. (b) Joined phylogenetic tree of moso bamboo *PheMADS14* (PH01000222G1190) and MADS transcription factors of *Arabidopsis* and *O. sativa*.

To explore the phylogenetic relationships among the *Dof* genes in moso, *Arabidopsis* and rice, we used MEGA 4.1 to construct a phylogenetic tree using full‐length CDS of *PheDof1* (918 bp, encoding 305 amino acids and 33.66 kD (Figure 4a). *PheDof1* (PH01000664G0640) was clustered in the same group with *OsDof1* and *OsDof29*, indicating that *PheDof1* was a member of the MCOG C subfamily.

We also constructed a phylogenetic tree to explore the relationship of *PheMADS14* with *Arabidopsis* and rice MADS‐box genes. The full‐length CDS of *PheMADS14* was 1077 bp, encoding 358 amino acids and a molecular weight of 39.49 kD (Figure 4b). *PheMADS14* (PH01000222G1190) was clustered in the same group as *OsMADS14*,* OsMADS15* and *OsMADS18*. Thus, *PheMADS14* was a member of the AP/AGL9 subfamily and participated in the formation of floral organs.

**Figure 5:**
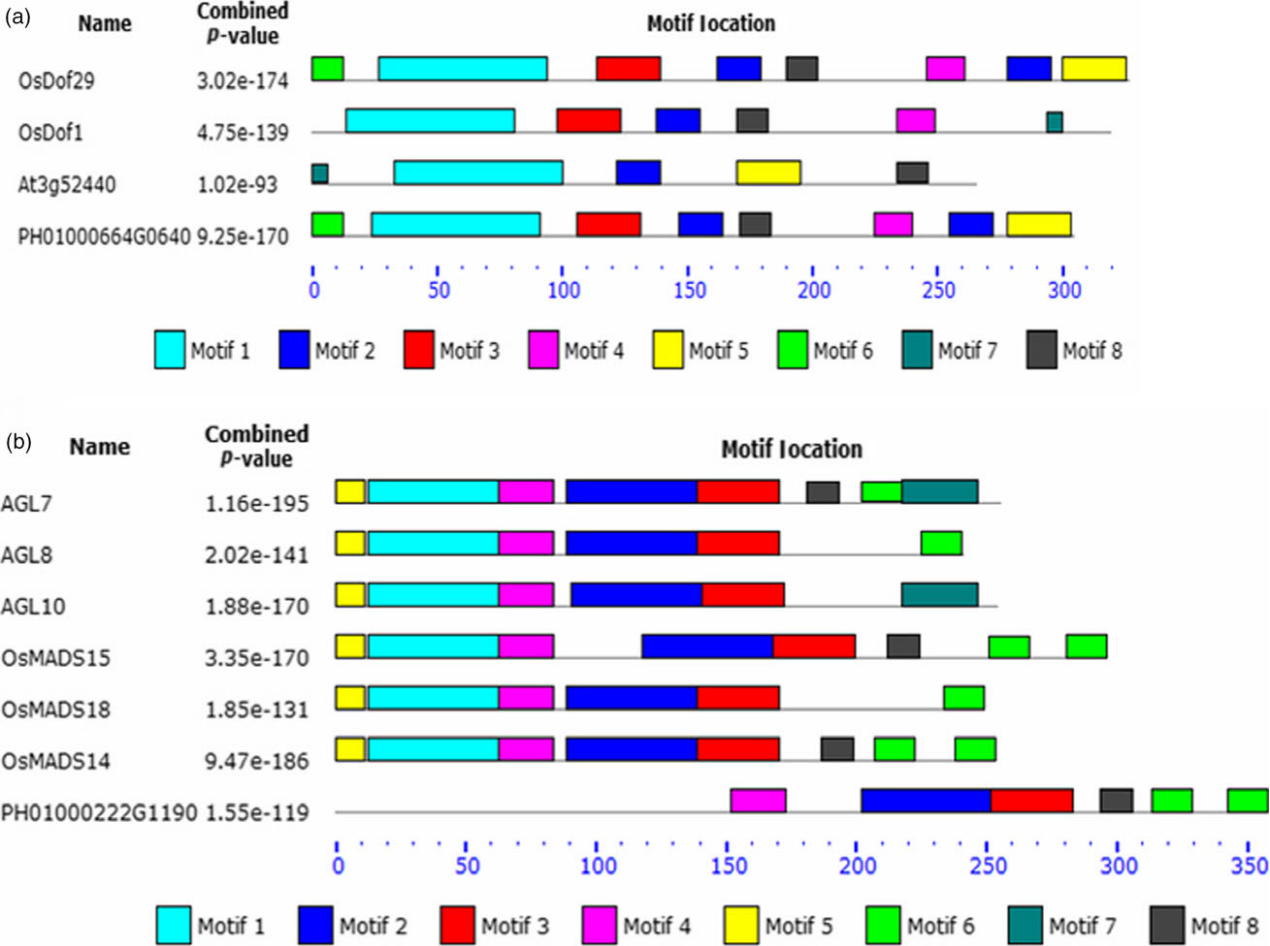
Distribution of the conserved motifs along the Dof and MADS transcription factors using the MEME software. (a) Dof transcription factors. (b) MADS transcription factors.

Protein sequence motifs analysis showed that *PheDof1* (PH01000664G0640) and *PheMADS14* (PH01000222G1190) belonged to the MCOG C and AP/AGL9 subfamilies, respectively (Figure 5a and b), which was consistent with our results of the MEGA4.1 analysis.

### Identification of PheDof1 and PheMADS14

**Figure 6:**
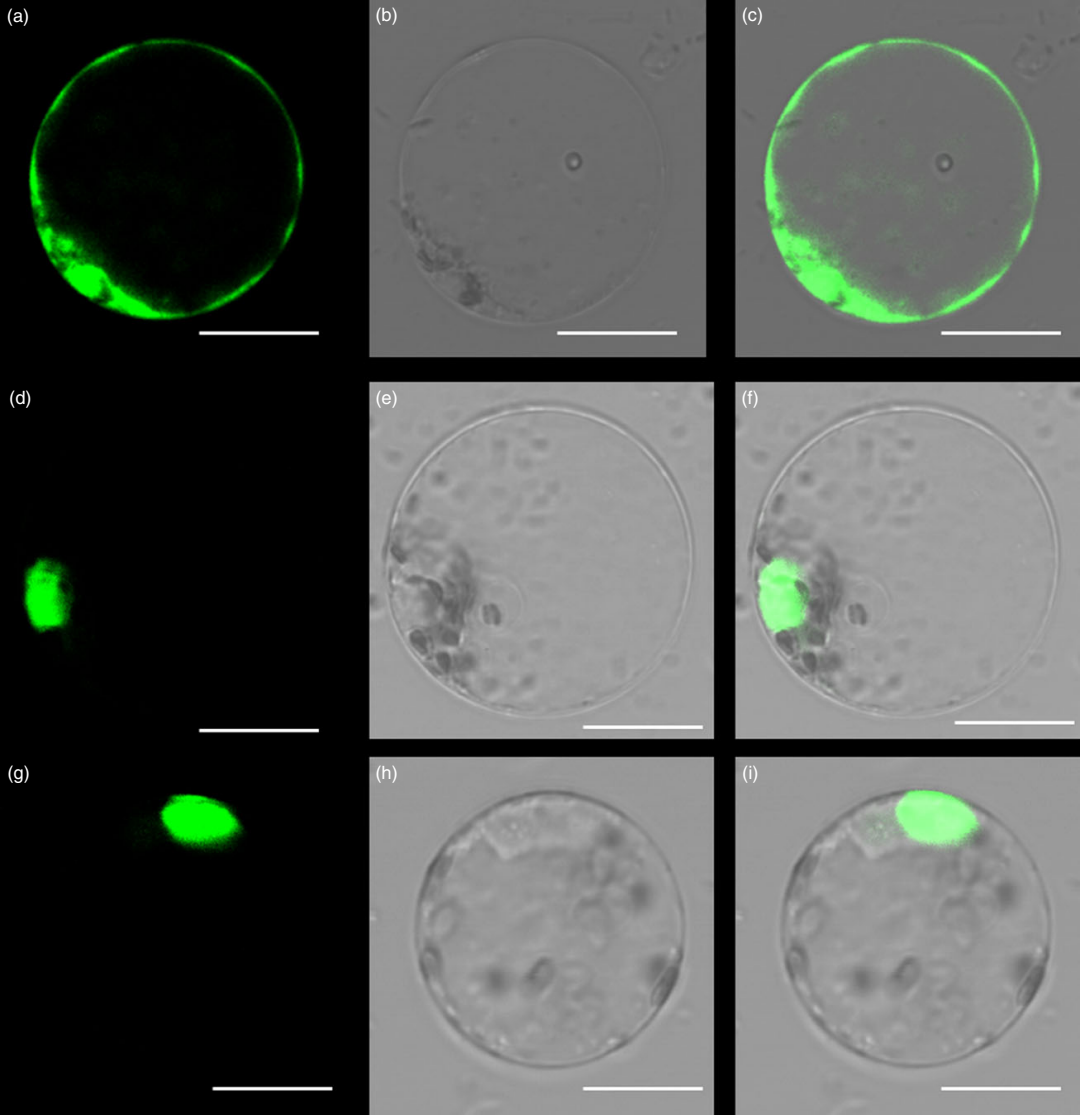
Subcellular localization of PheDof1 and PheMADS14. (a–c) Rice protoplasts expressing 35S‐GFP. (d–f) Rice protoplasts expressing PheDof1‐GFP. (g–i) Rice protoplasts expressing PheMADS14‐GFP. Bar = 10 μm.

**Figure 7:**
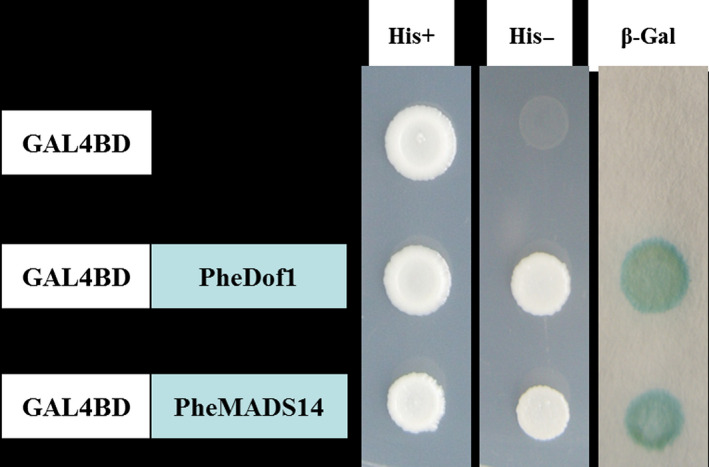
Transcriptional activation analysis of PheDof1 and PheMADS14.

The fluorescent protein‐tagging method was used to investigate whether PheDof1 and PheMADS14 were nuclear‐localized proteins like the transcription factors. The results showed that while green fluorescent protein (GFP) alone presented a dispersed cytoplasmic distribution, GFP‐tagged *PheDof1* and *PheMADS14* were located in the nucleus as per their functions as transcription factors (Figure 6). To further ascertain whether they were able to activate transcription, PheDof1 and PheMADS14 were each fused with GAL4 DNA‐binding domain (GAL4DB) and tested in yeast using a reporter construct to study whether PheDof1 and PheMADS14 had transcriptional activation activity. It was found that PheDof1 and PheMADS14 could activate the expression of the His‐3 and β‐Gal reporter genes (Figure 7), indicating that they are transcriptional activators.

### Western blot

**Figure 8:**
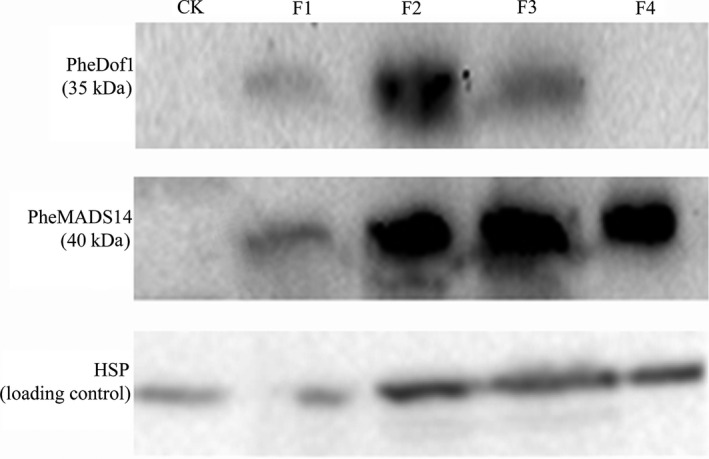
Western blot analysis of PheDof1 protein and PheMADS14 protein. HSP was used as the loading control. CK: nonflowering moso bamboo leaves, F1: floral bud formation stage, F2: inflorescence growing stage, F3: bloom stage, F4: embryo formation stage.

Western blots performed using floral buds, whole inflorescences and full blooms showed the presence of PheDof1 (35 kD) while no PheDof1 signal was detected in nonflowering leaves and embryo formation stages (Figure 8). PheMADS14 (40 kD) signal was detected at four different flower development stages. The signal was weak at the floral bud formation stage and relatively deep with the flower development. However, no PheMADS14 signal was detected in nonflowering leaves (Figure 8).

### Expression patterns of PheDof1 and PheMADS14

**Figure 9:**
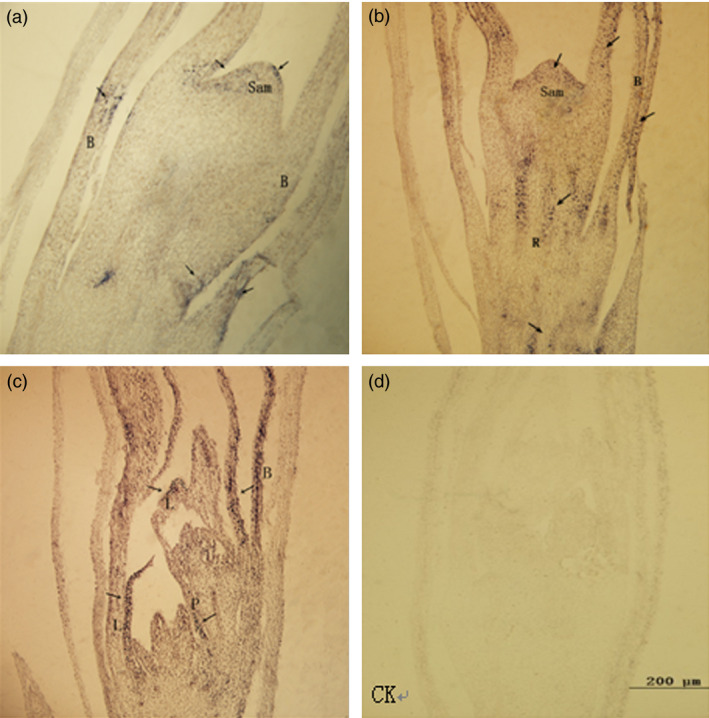
The expression of *PheDof1* in moso bamboo flower formation of different developmental periods. B = bract, R = rachis, Sam = shoot apical meristem, L = lemma, P = Palea. (a–b) The floral bud formation stage. (c) Bloom stage. (d) The expression of sense probe after *in situ* hybridization.

Antisense RNA probes of *PheDof1* and *PheMADS14* were used to perform *in situ* hybridization on paraffin sections of moso bamboo flowers at different developmental stages to explore the temporal and spatial expression patterns of *PheDof1* and *PheMADS14* in the process of moso bamboo flower development. Our results showed that *PheDof1* was expressed at the early stages of moso bamboo flower development and was strongly expressed in the apical growth cone and inflorescence axis at the floral bud formation stage (Figure 9). Moreover, *PheDof1* expression signals were also observed in bracts. Glumes and paleas were formed after the formation of pistil and stamen primordia. *PheDof1* was positively expressed in glumes and bracts primarily while almost no expression signal was observed in stamen and pistil primordia.

**Figure 10:**
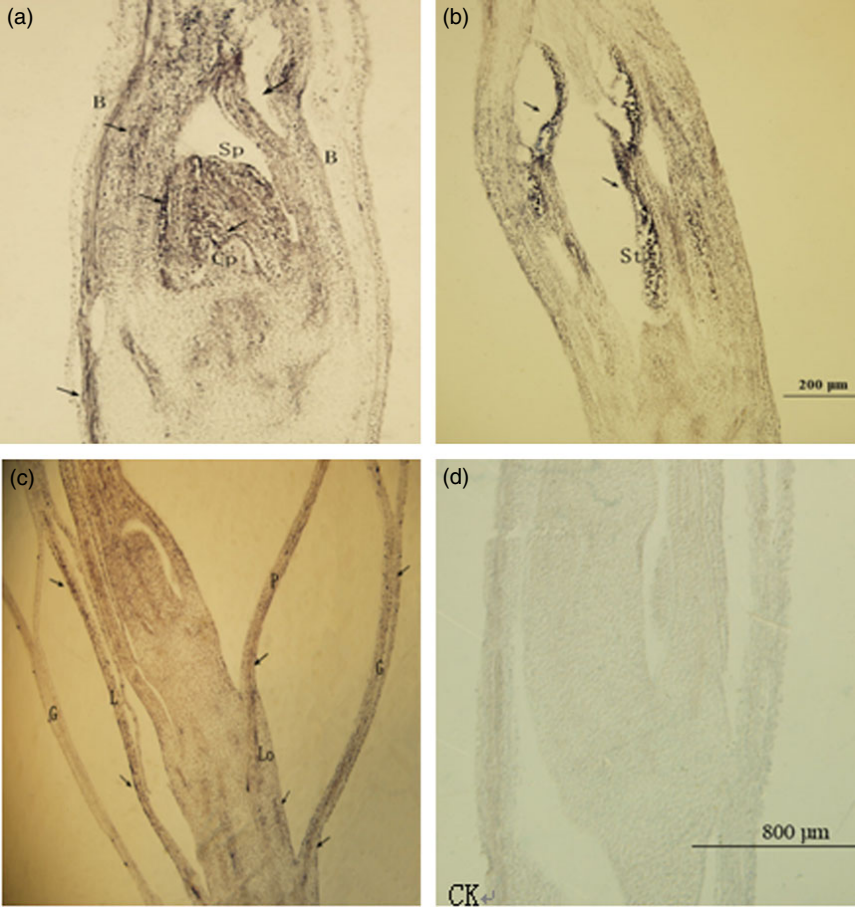
The expression of *PheMADS14* in moso bamboo flower formation of different developmental periods. G = glume, L = lemma, P = Palea, Lo = lodicule, B = bract, Cp = calyx primordia, Sp = stamen primordia, St = stamen. (a) Inflorescence growing stage. (b) Bloom stage. (c) Embryo formation stage. (d) The expression of sense probe after *in situ* hybridization.

*PheMADS14* was expressed in different parts of moso bamboo flowers and was mainly concentrated in the parts with strong cell division such as pollen tube, bracts, pistil and stamen primordia (Figure 10). Strong expression of *PheMADS14* was observed during the differentiation of pistil and stamen primordia, which was the rapidly proliferating stage of pistil and stamen cells. *PheMADS14* was strongly expressed in stamens while no expression signal was detected in pistils, embryos and endosperms. In the process of embryo formation, the *PheMADS14* expression was detected only in glumes, paleas and lodicules. These results suggest that *PheMADS14* plays significant roles in the processes of pistil and stamen primordia formation, but is not involved in embryo formation of moso bamboo.

### Expression analyses of miRNAs and target genes

**Figure 11:**
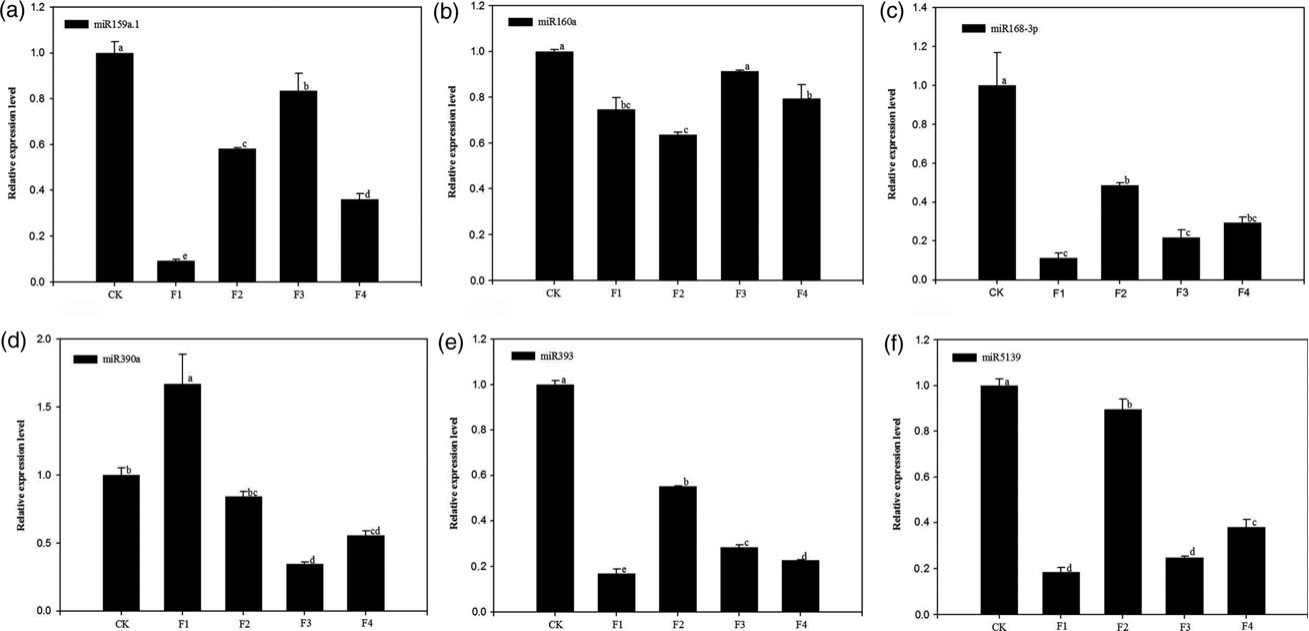
Expression patterns of 6 selected miRNAs among nonflowering leaves (CK) and different flower developmental stages (F1, F2, F3 and F4) in moso bamboo as determined by qRT‐PCR. U6 snRNA was used as a reference in qRT‐PCR. The level of every miRNA in the control was set at 1.0. Bars represent mean values of three replicates ± standard error. (a) Expression patterns of miR159a.1. (b) Expression patterns of miR160a. (c) Expression patterns of miR168‐3p. (d) Expression patterns of miR390a. (e) Expression patterns of miR393. (f) Expression patterns of miR5139.

Six miRNAs, miR159a.1, miR160a, miR168‐3p, miR390a, miR393 and miR5139 were selected for qRT‐PCR validation experiments (Figure 11). The miRNAs showed significant down‐regulation that progressed through the flowering stages in the nonflowering samples, which was consistent with our sequencing data. The six miRNAs possibly play vital regulatory roles in the moso bamboo blooming process especially the vegetative growth stage. We speculate that miR390a and miR5139 may be involved in plant–pathogen interactions while miR160a and miR393 may participate in plant hormone signal transduction. Besides, the significantly down‐regulated expressions of miR159a.1 and miR168‐3p indicated that they might play vital regulatory roles in protein processing in the endoplasmic reticulum.

**Figure 12:**
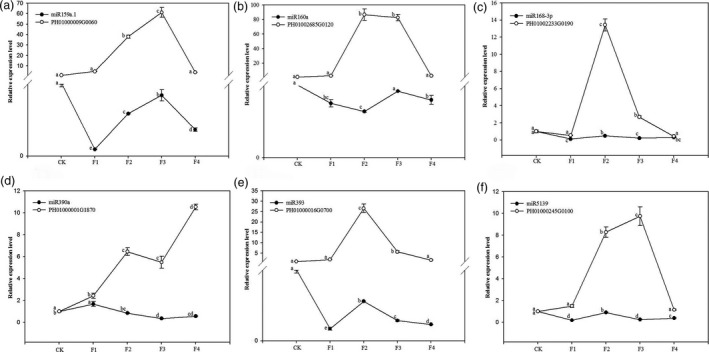
Expression patterns of 6 target genes and their corresponding miRNAs among nonflowering leaves (CK) and different flower developmental stages (F1, F2, F3 and F4) in moso bamboo as determined by qRT‐PCR. *TIP41* was chosen as the internal housekeeping gene control for target genes. The level of every gene in the control was set at 1.0. Bars represent mean values of three replicates ± standard error. (a) Expression patterns of PH01000009G0060 and miR159a.1. (b) Expression patterns of PH01002685G0120 and miR160a. (c) Expression patterns of PH01002233G0190 and miR168‐3p. (d) Expression patterns of PH01000001G1870 and miR390a. (e) Expression patterns of PH01000016G0700 and miR393. (f) Expression patterns of PH01000245G0100 and miR5139.

We measured the expression levels of six targets: PH01000009G0060 (MYB family transcription factor, miR159a.1 target), PH01002685G0120 (auxin response factor, miR160a target), PH01002233G0190 (DEK C‐terminal domain containing protein, miR168‐3p target), PH01000001G1870 (leucine‐rich repeat family protein, miR390a target), PH01000016G0700 (OsFBL16‐F‐box domain and LRR containing protein, miR393 target) and PH01000245G0100 (geminivirus Rep‐interacting motor protein, miR5139 target). These miR targets were measured during the development of moso bamboo flowers to verify that the target genes were actually regulated by the corresponding miRNAs. The relative expression levels of PH01000009G0060, PH01002685G0120, PH01002233G0190, PH01000001G1870, PH01000016G0700 and PH01000245G0100 increased from the nonflowering samples to the flowering samples; however, those of miR159a.1, miR160a, miR168‐3p, miR390a, miR393 and miR5139 were inhibited (Figure 12). The results indicated that the variation in miRNAs abundance had a negative effect on the abundance of their target genes. Thus, the expression profiles of miRNAs and their target genes were complementary.

### Overexpression of pre‐miR164a in Arabidopsis plants (wild‐type) delayed flowering time

**Figure 13:**
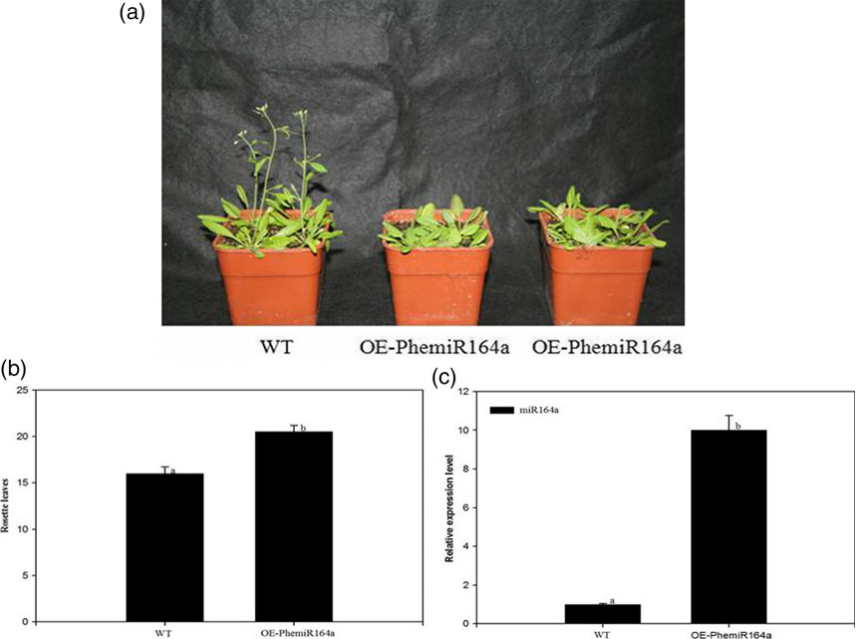
Analysis of a delayed flowering phenotype by overexpression of PhemiR164a in *Arabidopsis*. (a) The flowering phenotype of wild‐type and transgenic plants at 23 °C under long‐day conditions. (b) Flowering time of wild‐type and transgenic plants at 23 °C under long‐day conditions. (c) Relative expression of PhemiR164a in wild‐type and transgenic plants were elevated by qRT‐PCR. Error bars indicated the standard deviation.

As miR164a might be involved in protein processing in the endoplasmic reticulum and predicted to target NAC transcription factor indicating its role in the regulation of flowering process, we overexpressed the pre‐miR164a in *Arabidopsis* WT. The resulting transgenic plants showed a delayed flowering phenotype (Figure 13a and b). We further investigated its expression in the T_3_ generation to ascertain the effects of this construct (Figure 13c).

## Discussion

We showed that 11 703 differentially expressed genes changed significantly during the four flower development. They were also annotated with KEGG pathway and found to be involved in 128 metabolic pathways. Using cluster analysis, we identified two major groups: the up‐regulated group and the down‐regulated group. Moreover, 13 051 differentially expressed genes were found between different flower organs and nonflowering leaves of moso bamboo (unpublished data), indicating the reproducibility of the two group of sequencing data. The expression levels of genes related to glycometabolism, photosynthesis and nitrogenous metabolism pathways showed a down‐regulated trend in the process of flower development. Previous studies showed that nitrogen content and nutritional ingredients gradually decreased in the leaves, stems, roots and other organs with the flowering process (Gao *et al*., 2002; Zhan and Li, 2007). Plant flowering is believed to be an ageing phenomenon in plants where the nutrient consumption is increased while the ability to synthesize energy materials decreases. Therefore, the growth is inhibited and ‘hunger death’ appears, resulting in a large number of deaths after the flowering of moso bamboo (Chai *et al*., 2006). Moreover, the expressions of genes related to auxin (IAA) and cytokinin (CTK) synthesis are significantly down‐regulated during flower development consistent with the withering leaves and dried culms dried, whereas many genes such as *PheTFL1* related to moso bamboo flowering exhibited high expression levels in inflorescence and inflorescence axis (Qi *et al*., 2013a,b). Besides, the content of IAA at bloom stage significantly decreased while GA_3_ presented an opposite trend (Qi *et al*., 2013a,b). The highest expressional pathways in the up‐regulated groups are plant–pathogen interaction, plant hormone signal transduction and protein processing in the endoplasmic reticulum, which suggests that related genes play significant roles in moso bamboo flower formation and development.

A large number of transcription factors participate in the pathways of plant–pathogen interaction and protein processing in the endoplasmic reticulum. Hundreds of transcription factors have been isolated from advanced plants. They play important roles in plant growth, morphogenesis and resistance to environmental stress. Peng *et al*. (2013) implicated stress such as drought as the main cause of moso bamboo flowering. Our DGE analysis showed that 766 transcription factors were highly expressed in the moso bamboo flower formation and development, including WRKY (110), MADS‐Box (38), bHLH (34), Dof (15), NAC (61), HSF (4), HMG (4), bZIP (6), HSP20 (28), HSP70 (13), HSP90 (3), MYB (143), AP2/DREBP (32), RAD23 (14), GTA (46), CML (65), PR1 (11), RPS2 (35), RPS5 (9), FLS2 (41), PBS1 (31) and BAK1 (23). These transcription factors could play vital roles in the flower formation and development process of moso bamboo.

Plant hormones participate in all stages of plant growth and development (Richards *et al*., 2001). Putative homologs of gibberellin (GA) signalling pathway genes are highly expressed, including the gibberellin response modulator (GAMYB) and gibberellin receptors (*GID1*,* GID2*). They lead to the degradation of DELLAs and up‐regulation of *MYB21*,* MYB24* and *MYB57*, which are important for stamen filament growth (Alexandrov *et al*., 2009; Tsuji *et al*., 2006). High expression levels of these genes suggest that GA may play crucial roles in flower formation and development in moso bamboo. Jasmonic acid (JA) not only helps plants resist pathogens, but also regulates plant growth and development in all aspects including the flower formation and development, and fruit ripening (Oh *et al*., 2013). JA also plays significant roles in regulating flower morphogenesis. A synthetic gene mutant of JA could induce flowering in *O. sativa*,* Triticum aestivum* and *Sorghum bicolor* (Scott *et al*., 2004; Stintzi and Browse, 2000). Besides, GA could improve the activity of JA, resulting in the up‐regulation expression of MYB to promote stamen filament growth (Cheng *et al*., 2009). According to DGEs analysis, the up‐regulation of JA like genes in moso bamboo indicated that JA could interact with other hormones to jointly regulate moso bamboo flowering.

**Figure 14:**
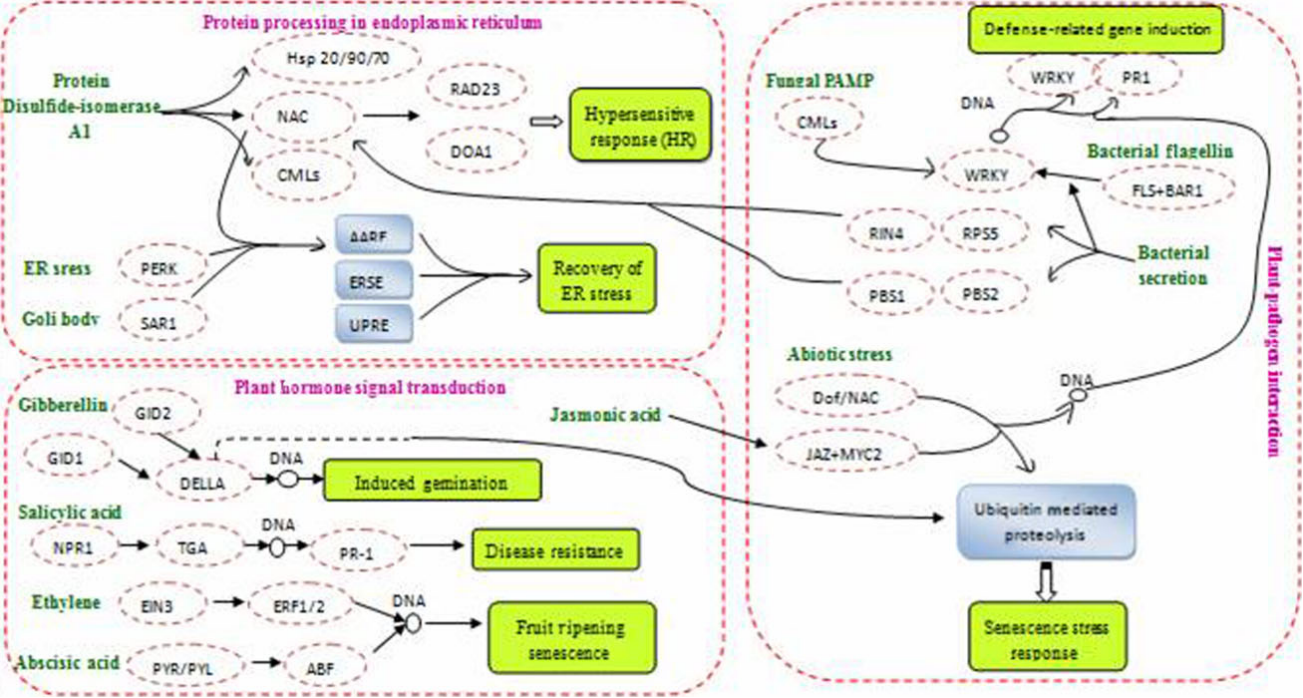
Different genes express in plant–pathogen interaction, plant hormone signal transduction and protein processing in endoplasmic reticulum pathways.

Plant–pathogen interaction, plant hormone signal transduction and protein processing in endoplasmic reticulum interact with each other and function together to regulate moso bamboo flowering (Figure 14). The related transcription factors of stress and hormone signal transduction genes identified in our study may be significant regulation factors in flower formation and development in moso bamboo. Research on the expression and regulation of these factors can help identify key genes involved in flower formation and development.

Dof (DNA binding with one zinc finger) transcription factors are a group of plant‐specific transcription factors containing a single Cys2/Cys2‐type zinc‐finger‐like Dof motif (Yanagisawa, 2002) that function in regulating flowering in response to photoperiod (Fornara *et al*., 2009). Ahmad *et al*. (2013) had found that the overexpression of *AtDof4.1* could delay the flowering time of *Arabidopsis* and affect the development of floral organs. Besides, *AtDof1.5* participated in the signal transduction pathway of phytochrome (Park *et al*., 2003) while *AtDof5.2, AtDof5.5* and *AtDof3.3* function in the photoperiod pathway (Imaizumi *et al*., 2005). Putative Dof transcription factors were detected in panicles, which were homologs of *Dof3*,* Dof4*,* Dof5*,* Dof12* and *CDF* (Cycling DOF Factor) family, revealing similar roles of *Dof3* and *Dof12* genes during the flower. Our sequence analysis studies showed that *PheDof1* was clustered with *OsDof1* and *OsDof29* and belonged to the MCOG C subfamily. We found *PheDof1* was located in the nucleus through the subcellular localization. PheDof1 was detected at early stages of flower formation and development, indicating that *PheDof1* might play a more significant role in the floral bud formation, inflorescence growth and bloom stages of moso bamboo. The strong expression of *PheDof1* was found in the apical growth cone and inflorescence axis at the floral bud formation stage through *in situ* hybridization, representing that *PheDof1* might take part in the start‐up of moso bamboo flowering. Gao *et al*. (2014) had identified *Hd3a*, a master floral developmental regulator in rice (with apparent paralogous counterparts in *Arabidopsis* called *FT*). Drought or other environmental stresses could regulate *MADS14* in the flowering stage of bamboo (Peng *et al*., 2013). Thus, a regulatory pathway of Dof‐Hd3a‐MADS‐flowering could play a vital role in moso bamboo flowering.

MADS‐box transcription factors contain a DNA‐binding domain conserved among eukaryotes (Airoldi and Davies, 2012). Many MADS family members had been described in model plants with the floral organ identity and development proteins falling into the MIKC clade such as *MADS5*,* MADS14* and *MADS15* (Beth and Jennifer, 2005; Parenicova *et al*., 2003; Theiben, 2001), which played significant roles in flower morphogenesis. *OsMADS14*,* OsMADS15* and *OsMADS18* belong to AP/AGL9 family, the expression patterns of which are similar to those of AP1 in *Arabidopsis*. They could determine the characteristics of floral organ meristem and are mainly expressed in glumes, paleas and lodicule primordia of *O. sativa* (Shinozuka *et al*., 1999). Kyozuka *et al*. (2000) showed the expression of *OsMADS15* in the apical meristem at the early development of spikelets and found it gradually located in lodicules and paleas with spikelet differentiation. *OsMADS14* is mainly expressed in glumes and paleas at the early spikelet development stage. When spikelets become mature, *OsMADS14* accumulates in stamens and carpels (Jeon *et al*., 2000; Pelucchi *et al*., 2002). Thirty‐eight MADS‐box transcription factors were identified in moso bamboo flowers, which were homologs of *MADS1*,* MADS2*,* MADS4*,* MADS7*,* MADS11*,* MADS14*,* MADS15*,* MADS17*,* MADS27*,* MADS31* and *MADS58* from *Z. mays*,* O. sativa* and *Brachypodium distachyon* (Gao *et al*., 2014). *PheMADS14* was clustered with *OsMADS14*,* OsMADS15* and *OsMADS18* and grouped in the AP/AGL9 subfamily. We found *PheMADS14* was also located in the nucleus through the subcellular localization. PheMADS14 exhibited high expressions at later stages of flower formation and development, while a lower expression at the floral bud formation stage. Strong expression signals of *PheMADS14* were detected in pistil and stamen primordia through *in situ* hybridization, indicating that *PheMADS14* participated in the flower morphogenesis of moso bamboo and could play significant regulatory roles in moso bamboo flowering.

miRNAs function in regulating almost all aspects of plant growth and development. Our study showed that miR159a.1, miR160a, miR168‐3p, miR390a, miR393 and miR5139 were involved in plant–pathogen interaction, plant hormone signal transduction and protein processing in endoplasmic reticulum based on the DGE analysis of their targets. They showed differential expression patterns from nonflowering leaves to flowering samples. Cluster analysis indicated the six miRNAs belonged to a down‐regulated group. The differential expression patterns of miRNAs, rather than the miRNA composition, might play greater roles in regulating moso bamboo blossom. The expression patterns of miRNAs in moso bamboo could provide significant clues to their biological function and regulatory mechanism. We further corroborated the relative expression levels of six selected miRNAs through qRT‐PCR. The results showed that relative expression levels of miR159a.1, miR160a, miR168‐3p, miR390a, miR393 and miR5139 in nonflowering leaves were higher than those of the four flowering samples indicating their roles in moso bamboo flowering. miR390a and miR5139 may be involved in plant–pathogen interaction. miR390a could inhibit the expressions of *ARF2*/*ARF3*/*ARF4* by regulating target genes and cause a series of phenotypic changes. miR390a plays an important regulatory role in organs development (Garcia *et al*., 2006) and growth cycle transition (Cho *et al*., 2012). The over expression of miR390a may affect the transition from vegetative growth to reproductive growth. The target of miR5139 is geminivirus Rep‐interacting motor protein. The expression of miR5139 is down‐regulated under stress, so moso bamboo flowering may be related to stress. miR160a and miR393 could play potential vital roles in plant hormone signal transduction. The targets of miR160a are ARF (Rhoades *et al*., 2002). miR160a participates in auxin signal transduction by targeting ARF and affecting vegetative growth and reproductive growth of plants (Mallory *et al*., 2005). The targets of miR393 belong to F‐box family (Navarro *et al*., 2006), and it regulates the plant perception to auxin increasing auxin sensitivity (Parry *et al*., 2009). miR159a.1 and miR168‐3p may participate in protein processing in the endoplasmic reticulum. miR159a.1 mainly regulates MYB and directly degrades *MYB* mRNA (Patrick *et al*., 2004), leading to a flowering delay. miR168 is important for the differentiation of plant tissues and the response to stress. Moreover, miR168 is the only miRNA that can be determined to be involved in the cross‐border regulation of animals and plants (Zhang *et al*., 2012). Thus, the over expressions of miR159a.1, miR160a, miR168‐3p, miR390a, miR393 and miR5139 might inhibit moso bamboo flowering. Our study showed that their relative expressions were down‐regulated during the moso bamboo flowering period. Our research indicated that the expression profiles of miRNAs were negatively correlated with those of their targets respectively, which further validated the regulation roles of miRNAs on their target genes and moso bamboo flowering.

miR164a targets *CUC2* which belongs to the NAC family and prompts the development of floral organ (Takada *et al*., 2001). miR164a negatively regulates *CUC1* and *CUC2* to inhibit the meristem formation and floral development, influencing the establishment of organ primordia boundary (Mallory *et al*., 2004). miR164a could participate in protein processing in the endoplasmic reticulum and affect plant flowering by regulating flower development. We report the isolation and characterization of pre‐miR164a from moso bamboo. In our research, the phenotype of delaying flowering time was also observed in pCAMBIA2300‐miR164a transgenic plants. The expression level of miR164a was up‐regulated in transgenic plants as compared with WT plants. The results led us to suspect that miR164a delayed flowering time by regulating NAC in transgenic *Arabidopsis*. Kim *et al*. (2009) thought that miR164 could delay plant senescence. Our findings suggested that miR164a might play significant roles in moso bamboo flowering.

We selected significant regulatory pathways and analysed with miRNAs at different flower developmental stages of moso bamboo. Some candidate genes involved in plant–pathogen interaction, plant hormone signal transduction and protein processing in endoplasmic reticulum were selected and investigated. Our findings indicated that *PheDof1*,* PheMADS14* and several miRNAs might be significant regulators in moso bamboo flowering and flower development. The study will provide vital information for further functional research and key genes selection and help to explore the molecular mechanism in moso bamboo flowering and senescence.

## Experimental procedures

### Plant materials

Flowering moso bamboo samples at different stages and nonflowering moso bamboo leaves (CK) were collected from Guilin (E 110°17′–110°47′; E 25°04′–25°48′) in the Guangxi Zhuang Autonomous Region. The samples were stored in stationary FAA (18: 1: 1 Formaldehyde, Glacial Acetic acid and 70% alcohol). The four stages were defined according to the anatomical structure of floral organs: F1 (floral bud formation, during which a plant transits from vegetative stage to reproductive stage), F2 (inflorescence axis continued to stretch, and lateral buds started to differentiate), F3 (bloom stage, flowers with both pistils and stamens emerging from glumes), F4 (embryo formation) (Gao *et al*., 2015). Besides the above, a large number of flowering moso bamboo samples at different stages and nonflowering moso bamboo samples were flash frozen in liquid nitrogen and stored at −80 °C to do further study.

### DGEs analysis and small RNA analysis

Tag library preparation for samples of the four different flowering developmental periods (F1, F2, F3 and F4) was performed in parallel using the Illumina gene expression sample preparation kit. Transcriptome data of nonflowering moso bamboo leaves (CK) were regarded as the reference gene database, and the four libraries were sequenced using the Illumina high‐seq 2000 at Beijing Genomics Institute (BGI) (Shenzhen, Guangdong Province, China) (Gao *et al*., 2014).

Total RNA was extracted from the frozen samples (CK, F1, F2, F3 and F4) using the Trizol reagent (Invitrogen, Carlsbad, California, USA), according to the manufacturer's instructions. Five small RNA libraries were constructed for moso bamboo, and the Illumina high‐seq 2000 sequencing was carried out by the Beijing Genomics Institute (BGI) (Shenzhen, Guangdong Province, China) (Gao *et al*., 2015).

KEGG pathway analysis was performed after we obtained the sequencing data. Key regulation pathways and genes were selected according to expression abundance. Hierarchical clustering of expressional data was carried out using the Cluster 3.0 and Treeview programs (Eisen *et al*., 1998).

### Bioinformatics analysis of PheDof1 and PheMADS14

The Dof and MADS transcription factors of moso bamboo, *Arabidopsis* and *Oryza sativa* were used to construct phylogenetic tree through MEGA 4.1. We used MEME 4.9 (http://nbcr-222.ucsd.edu/opal-jobs/) to determine the conserved motifs of protein sequence.

### Subcellular localization and transcriptional activation

The subcellular localization of PheDof1 and PheMADS14 was determined by transfecting GFP‐tagged PheDof1 and PheMADS14 into rice stem and sheath protoplasts (Zhang *et al*., 2011). The full‐length cDNAs of *PheDof1* and *PheMADS14* were fused in frame with the GFP cDNA and ligated between the CaMV 35S promoter and the nopaline synthase terminator. The fluorescence signals in transfected protoplasts were examined using a confocal laser scanning microscope (Leica Microsystems).

The transcriptional activation activity of PheDof1 and PheMADS14 was studied by transforming the pGBKT7 construct (that had the fusion of these genes and the GAL4 DNA‐binding domain) into the yeast strain PJ. The yeast strain contained the *His‐3* and *LacZ* reporter genes. The transformed yeast cells were grown on synthetic defined plates (with or without *His*) and were assayed for β‐galactosidase activity.

### Western blotting

To generate antibodies against PheDof1 and PheMADS14, we purified the two fusion proteins from *E. coli* and used them to immunize a New Zealand white rabbit. The PheDof1 and PheMADS14 antibodies were immunopurified against nitrocellulose‐bound antigen. We separated the 20 μg crude, soluble extract on 5%–10% polyacrylamide gels followed by blotting onto nitrocellulose membrane. Blots were incubated with affinity‐purified PheDof1 and PheMADS14 antibodies followed by goat anti‐rabbit IgG‐HRP (Sigma) and detected by enhanced chemiluminescence using Super Signal West Fempto Maximum Sensitivity Substrate (Thermo Scientific).

### *In situ* hybridization

*In situ* hybridization was performed as previously described (Hord *et al*., 2006). Flowering moso bamboo samples at different stages were fixed in the stationary liquid, FAA (18: 1: 1 Formaldehyde, Glacial Acetic acid and 70% alcohol). The samples were fixed for 48 h, dehydrated and embedded in paraffin, and 10‐μm‐thick sections were prepared with microtome (Leica, Germany) and mounted onto slides. The slides were dehydrated and baked, followed by dewaxing of all slides with dimethylbenzene. The antisense and sense RNAs of *PheDof1* and *PheMADS14* were labelled with digoxigenin by *in vitro* transcription of linearized pGEM‐T‐PheDof1 and pGEM‐T‐PheMADS14, which carried fragments of the *PheDof1* cDNA amplified with gene‐specific primers YWZJ‐Dof‐F (5′‐GCTGGCGAGCACAGAGGT) and YWZJ‐Dof‐R (5′‐CGCTGCTCCAAACATCG), and *PheMADS14* cDNA amplified with gene‐specific primers YWZJ‐MADS‐F (5′‐AGGTAAAGTTTGTAAGTGGGC) and YWZJ‐MADS‐R (5′‐AGTTGCTGCTCTAGCTGCTGA). Antidigoxigenin antibodies coupled with alkaline phosphatase and nitro blue tetrazolium were used to detect the hybridization signal.

### Quantitative real‐time PCR (qRT‐PCR) analysis of miRNAs and target genes in moso bamboo

Total RNA was extracted from the frozen samples (CK, F1, F2, F3 and F4) and used for cDNA synthesis using the miRNA‐specific stem–loop RT primer. The reaction was incubated at 16 °C for 30 min, 42 °C for 30 min, 85 °C for 5 min and 4 °C for 5 min. Real‐time PCR was carried out on a Light Cycler 480 machine (Roche) using a SYBR Green I Master Kit (Roche, Switzerland). The final volume was 20 μL, containing 10 μL 2 × SYBR Premix Ex Taq, 7.2 μL of nuclease‐free water, 0.4 μL of each primer (10 μm) and 2 μL of cDNA. The amplification was carried out as follows: initial denaturation at 95 °C for 10 min, followed by 43 cycles at 95 °C for 10 s, 58 °C for 20 s and 72 °C for 10 s. The melting curves were adjusted as 95 °C for 5 s and 58 °C for 1 min and then cooled to 40 °C for 30 s (Unver and Budak, 2009). All reactions were performed in triplicates. U6 snRNA were chosen as internal control for the miRNAs (Ding *et al*., 2011). The stem–loop reverse transcription primers were designed following the method described by Chen *et al*. (2005). PCR primers including a miRNA‐specific forward primer and a reverse primer were used to amplify the PCR products.

The expression analysis of several target genes was also examined by quantitative RT‐PCR. Reverse transcription reactions were performed using 2 μg of RNA by M‐MLVRT (Promega, Madison, Wisconsin, USA) according to the manufacturer's instructions. We obtained the sequences of four selected target genes from the moso bamboo genome database (http://www.ncgr.ac.cn/bamboo). We selected *TIP41* as the internal housekeeping gene control (Fan *et al*. 2013). Real‐time PCR was carried out on a Light Cycler 480 machine (Roche) using a SYBR Green I Master Kit (Roche, Switzerland). The 20 μL reaction mixture contained 10 μL 2 × SYBR Premix Ex Taq, 7.2 μL of nuclease‐free water, 0.4 μL of each primer (10 μm) and 2 μL of cDNA. Amplification reactions were performed as the following: 95 °C for 10 s, 60 °C for 10 s and 72 °C for 20 s. All reactions were carried out in triplicate. The primers used in all quantitative RT‐PCR experiments are listed in Table 1.

**Table 1:** Primers used in miRNAs and targets qRT‐PCR

Name	Primer	Sequence
miR159a.1	RT Primer	CTCAACTGGTGTCGTGGAGTCGGCAATTCAGTTGAGCAGAGCTC
	Forward Primer	ACGGGCTTTGGATTGAAGGGA
miR160a	RT Primer	CTCAACTGGTGTCGTGGAGTCGGCAATTCAGTTGAGTGGCATAC
	Forward Primer	AGCCCTGCCTGGCTCCCTGT
miR164a	RT Primer	CTCAACTGGTGTCGTGGAGTCGGCAATTCAGTTGAGTGCACGTG
	Forward Primer	AGCCCTGGAGAAGCAGGGCA
miR168‐3p	RT Primer	CTCAACTGGTGTCGTGGAGTCGGCAATTCAGTTGAGATTCACTT
	Forward Primer	AGCCCGCCCGCCTTGCACCAA
miR390a	RT Primer	CTCAACTGGTGTCGTGGAGTCGGCAATTCAGTTGAGGGCGCTAT
	Forward Primer	ACGGGAAGCTCAGGAGGGAT
miR393	RT Primer	CTCAACTGGTGTCGTGGAGTCGGCAATTCAGTTGAGATCAATGC
	Forward Primer	ACGGGCTCCAAAGGGATCGC
miR5139	RT Primer	CTCAACTGGTGTCGTGGAGTCGGCAATTCAGTTGAGTGGTATCA
	Forward Primer	ACGGGCAACCTGGCTCTGATA
	Universal Reverse Primer	CTCAACTGGTGTCGTGGAGTC
U6	Forward Primer	GGACATCCGATAAAATTGGAACGATACAG
	Reverse Primer	AATTTGGACCATTTCTCGATTTATGCGTGT
PH01000009G0060	Forward Primer	TTCACTGAATCCACCCCTCC
	Reverse Primer	ATGAGGTGAAGTGTCCCCAG
PH01002685G0120	Forward Primer	CTGCAGTCGAGCCTAGTGTA
	Reverse Primer	CAGACCGCCAGAATTGCTAC
PH01002233G0190	Forward Primer	ACAACTAGCAAGGGTCCACA
	Reverse Primer	TGATGATTCCCCTTTGCCCT
PH01000001G1870	Forward Primer	GTCCACCAACAGCGATGATC
	Reverse Primer	TTCTGCTCATCCCCACCATT
PH01000016G0700	Forward Primer	CGGGGAATTTGACGGATGAC
	Reverse Primer	ACGTTGGAGTTTCACACAGC
PH01000245G0100	Forward Primer	GTCCCCAGAACTTCAGTCCA
	Reverse Primer	TCCTCAATCCCACGAAGCTT
TIP41	Forward Primer	AAAATCATTGTAGGCCATTGTCG
	Reverse Primer	ACTAAATTAAGCCAGCGGGAGTG

### Constructs and plant transformation

For pCAMBIA2300‐miR164a construct, a fragment of pre‐miR164a from moso bamboo was obtained by PCR amplification using the pre‐miR164a‐F (5′‐TAGGATCCCAAACCGTGCTGGAGAAGC) and pre‐miR164a‐R (5′‐CGAAGCTTGAAGCCATGGTGGAGAAGGAG) primers. The construct was introduced into wild‐type *Arabidopsis* plants (Columbia‐0). Electroporation transformation was performed as described by *Agrobacterium*‐mediated transformation. Transgenic plants were selected on kanamycin, and the first generation of transgenic plants was examined for their phenotypes. At least 10 independent transgenic plants exhibiting severe phenotypes were selected for phenotypic characterization and examined.

## Conflict of interest

All the authors have declared no conflict of interest.
